# Modulatory role of atorvastatin against high-fat diet and zymosan-induced activation of TLR2/NF-ƙB signaling pathway in C57BL/6 mice

**DOI:** 10.22038/ijbms.2021.55460.12409

**Published:** 2021-08

**Authors:** Priyanka Arya, Sayima Nabi, Uma Bhandari

**Affiliations:** 1 Department of Pharmacology, School of Pharmaceutical Education & Research (SPER), Jamia Hamdard (UGC approved deemed to be University, Govt. of India), New Delhi- 110062, India

**Keywords:** Atorvastatin, High-fat diet, Inflammation, Low-density lipoprotein – receptor, Nuclear factor-kappa B, Toll-like receptor, Zymosan

## Abstract

**Objective(s):**

Accumulated evidence provides a strong connection between the immune system and vascular inflammation. The innate immune system’s main sensors are toll-like receptors (TLRs). Zymosan (Zym), a fungal product, induces an inflammatory response via activating TLR2 of the immune system. Atorvastatin, a potent statin, possesses pleiotropic effects including immunomodulatory, lipid-lowering, and anti-inflammatory. Therefore, the current study aimed to evaluate the protective role of atorvastatin against a high-fat diet (HFD) and Zym-induced vascular inflammation in C57BL/6 mice via modulation of TLR2/NF-ƙB signaling pathway.

**Materials and Methods:**

*In silico *study was conducted to confirm the binding affinity of atorvastatin against TLR2. Under *in vivo* study, mice were divided into four groups: Normal control: normal standard chow-diet fed for 30 days + Zym vehicle (sterile PBS, 5 mg/ml on 8^th^ day); HFD (30 days) + Zym (80 mg/kg, IP, on 8^th^ day); HFD/Zym + atorvastatin vehicle (0.5% CMC, p.o., from 10^th^ to 30^th^ day); HFD/Zym + atorvastatin (3.6 mg/kg, p.o., from 10^th^ to 30^th^ day).

**Results:**

Atorvastatin treatment along with HFD and Zym inhibited vascular inflammation by suppressing the levels of aortic TLR2, cardiac NF-ƙB and decrease in serum TNF-α and IL-6. Further, there was an increase in hepatic LDLR levels, resulting in a decrease in serum LDL-C and an increase in HDL-C levels. Histopathological examination of the aorta showed a reduction in plaque accumulation with the atorvastatin-treated group as compared with HFD and Zym-treated group.

**Conclusion:**

Atorvastatin attenuates vascular inflammation mediated by HFD and Zym through suppression of TLR2, NF-ƙB, TNF-α, IL-6, and upregulation of LDLR levels.

## Introduction

Vascular inflammatory disease is derived by the buildup of cells from both innate and acquired immune systems inside the intima of the artery wall, and it occurs at all phases of atherosclerotic disease progression, from the early development of lesion to the incidence of clinical events (1, 2). Toll-like receptors (TLRs) are the crucial part of immune systems that provide a strong connection between vascular inflammation, infection, and atherosclerosis development (3). TLRs are known as pattern recognition receptors, and their primary role is to recognize pathogens and mediate infection prevention. Activation of TLRs triggers the inflammatory signaling pathway and releases a variety of cytokines which affects vascular functions (4).

Increased intake of a high caloric diet alters adipose tissue lipolysis and lipid metabolism. It causes the release of inflammatory mediators, which contributes to the development of atherosclerosis (5). A previous study demonstrated that consumption of HFD for 4 weeks (28 days) in rats caused lipid dysregulation as well as enhanced systemic oxidative and inflammatory stresses in the heart (6). Zymosan A (Zym) a microbial substance derived from the yeast cell wall of *Saccharomyces cerevisiae,* has been used as an inflammatory agent (7, 8). Collected evidence suggested that Zym stimulates the immune system by activation of TLR2, and vascular inflammation in atherosclerosis is associated with the activation of Zym-induced TLR2 signaling pathways which then transfer transmembrane signals that activate nuclear factor-kappa B (NF-ƙB) (2, 9-10). It is a crucial transcription factor for the induction of inflammatory cytokines and also altered lipid metabolism by disruption of low-density lipoprotein receptor (LDLR); this causes foam cells to develop and plaque to build up in the artery wall (11). This, in turn, disturbs the blood flow of vascular arteries and increases the risk of atherosclerosis. Previous studies have shown that C57BL/6 mice were the most susceptible strain to develop vascular inflammation-associated atherosclerosis (12, 13). Therefore, HFD and Zym were utilized as an experimental model to produce vascular inflammation in C57BL/6 mice in the current investigation (14, 15). 

Atorvastatin is the most effective treatment for dyslipidemia and prevents cardiovascular disease by inhibiting 3-hydroxyl-3-methylglutaryl coenzyme A (HMG-CoA) reductase (16). In addition to the lipid-lowering effect, atorvastatin appears to have pleiotropic properties such as immunomodulatory, anti-oxidant, anti-proliferative, anti-platelet, and anti-inflammatory (17, 18). Previous research has shown that atorvastatin inhibits inflammatory angiogenesis through down-regulation of the VEGF/TGF pathway in the sponge implant mice (19). Bruder-Nascimento and colleagues reported that atorvastatin prevents aldosterone-induced vascular inflammation by reducing oxidative stress in rats (20). Accumulated evidence suggests that TLR signaling, particularly TLR2 or TLR4, appears to alter the risk of coronary artery disease in humans (21). Numerous preclinical and clinical studies have shown the protective effect of statin therapy against coronary artery disease, hyperlipidemia, chronic heart failure, and hypercholesterolemia via inhibiting TLRs and their downstream signaling pathway (21-25). A study (26) reported that 4-weeks administration of atorvastatin reduces TLR4 expression in human CD14^+^ monocytes cells in a dose-dependent manner through inhibition of protein prenylation, which reduces the LPS-stimulated inflammatory cytokine levels. However, the effect of atorvastatin in HFD and Zym-induced vascular inflammation via modulation of the TLR2/NF-ƙB signaling pathway has not been explored yet. Therefore, the current research work was designed to investigate the protective role of atorvastatin against HFD and Zym-induced vascular inflammation in C57BL/6 mice via modulation of the TLR2/NF-ƙB signaling pathway.

## Materials and Methods

***Drugs and chemicals***

Pinnacle Life Sciences, Pvt. Ltd, Himachal Pradesh, India, provided atorvastatin as a gift sample. Ashirwad Industries, Punjab, India, provided a gift sample of the HFD. The composition of the HFD used was 45% kcal fat, 35% kcal, and carbohydrates 20% kcal protein (27). The other chemicals used in the experimental study were purchased from Sigma Chemicals, St. Louis, Missouri, USA.

***In silico study (molecular docking)***

We performed molecular docking of atorvastatin at the TLR2 protein receptor-ligand binding site to gain a better understanding of the binding mode of atorvastatin at the molecular level. The docking of atorvastatin was carried out with Maestro, version 10.6 of the Schrodinger software suite. Using the build panel, the ligand was sketched in 3D format and prepared for docking using the LigPrep tool. The protein for the docking investigation was obtained from the Protein Data Bank (PDB ID: 3a7b) and prepared by removing the solvent, adding hydrogen, and further energy minimization using protein preparation wizard (28). The co-crystallized ligand, LTC** (**(2S)-1-({3-O-[2-(acetylamino)-4-amino-2,4,6-trideoxy-beta-D-galactopyranosyl]-alpha-D-glucopyranosyl}oxy)-3(heptanoyloxy)propan-2-yl (7Z)-pentadec-7-enoate**)** was used to create a grid for molecular docking in the protein (28). The docking was validated by withdrawing LTC from the site and then re-docked into the active site of the TLR2/TLR1 heterodimer. Atorvastatin was then docked at a similar site after validation.

***Animals***

The experimental research work was approved by the Institutional Animal Ethics Committee (IAEC) Jamia Hamdard, New Delhi, India, which was carried out in accordance with the Committee for the Purpose of Control and Supervision of Experiments on Animals (CPCSEA) guidelines (Registration no. of JHAEC: 173/GO/Re/S/2000/CPCSEA, Approved Protocol No. 1389, Approval date-18/09/2017). C57BL/6 mice were aged 9–13 weeks, obtained from the Central Animal House Facility of Jamia Hamdard, New Delhi, India. The mice were housed in polypropylene cages with a 12-hour light-dark cycle, a temperature of 22±2 °C, and relative humidity of 55±5% with food and water *ad libitum*.

***Induction of experimental vascular inflammation***

Zym (80 mg/kg) was dissolved in sterile phosphate-buffered saline solution (PBS) to a final concentration of 5 mg/ml (8). In C57BL/6 mice, vascular inflammation was induced by feeding pellets of HFD at random for 30 days, followed by a single intraperitoneal (IP) injection of Zym on the 8^th^ day**. **The dose of Zym (80 mg/kg, single *IP *injection) was selected based on a previous study (8, 14). An earlier study demonstrated that intake of HFD for 4 weeks (28 days) in rats caused lipid dysregulation as well as enhanced systemic oxidative and inflammatory stresses in the heart (6). Therefore in the current investigation, we selected the consumption of HFD in mice for 30 days along with Zym to induce vascular inflammation. 

***Experimental design***

Mice were randomly divided into four groups, each group consisting of six mice. Group I / Normal control: Mice were given regular standard chow-diet for 30 days and the sterile PBS (Zym vehicle) (80 mg/kg, *IP*, single injection) on the 8^th ^day; Group II / HFD + Zym: Mice were administered HFD for 30 days and Zym 80 mg/kg, *IP,* single injection on 8^th ^day; Group III / HFD/Zym + atorv vehicle: Mice were given HFD for 30 days and Zym (80 mg/kg, *IP,* single injection on 8^th ^day) + 0.5% carboxymethylcellulose (CMC) (atorvastatin vehicle, *p.o.*) from day 10^th ^ to 30^th ^day; Group IV / HFD/Zym + atorv (3.6): Mice were administered HFD for 30 days + Zym (80 mg/kg, *IP,* single injection on 8^th^ day) + atorvastatin (3.6 mg/kg/day*, p.o.)* from day 10^t^ to 30^th^ day (total 20 days treatment of atorvastatin). The atorvastatin dose was determined based upon previous research (19, 29). Blood was obtained from overnight fasting mice on the 31^ st^ day and centrifuged to separate serum, which was then stored at 20 °C for different lipids, interleukin-6 (IL-6), and tumor necrosis factor (TNF-α) assessments. The animals were sacrificed, and the aorta, heart, and liver were isolated to determine the levels of aortic TLR2, cardiac NF-ƙB, and liver LDLR. For histopathological study, a small portion of heart and aorta tissue was stored in a formalin solution (10%).

***Measurement of anthropometric parameters***

The difference between final and beginning body weight was used to calculate weekly body weight changes. The amount of food left in the cages subtracted from the total amount of food delivered to each mouse was used to determine their food consumption. The amount of water consumed was calculated by subtracting the amount of water left in the water bottle from the total amount of water given to each mouse (30).

***Measurement of serum lipids profile***

Total cholesterol (TC), triglycerides (TGs), and high-density lipoprotein-cholesterol (HDL-C) were tested in serum using commercial kits (Span Diagnostics Ltd, Surat, Gujarat, India) (HDL-C; Reckon Diagnostics Pvt Ltd, Baroda, Gujarat, India). Friedewald’s equation was used to calculate the levels of low-density lipoprotein-cholesterol (LDL-C) and very-low-density lipoprotein (VLDL): LDL-C =TC − HDL −VLDL; VLDL = TGs/5 (31-33). The atherogenic index (AI) and coronary risk index (CRI) were derived using the formulas LDL-C/HDL-C and TC/HDL-C, respectively (34).

***Assessment of aortic TLR2, *** liver*** LDLR, and serum TNF-α, IL-6 levels***

TLR2 levels in aortic tissue, LDLR protein in hepatic tissue, and TNF- and IL-6 levels in serum were determined using an ELISA kit as per the instructions recommended by the manufacturer (Genxbio Health Sciences Pvt. Ltd.) (35-38).

***Immunohistochemical (IHC) analysis of NF-*** ƙ***B of heart left ventricle (LV) tissue ***

The cardiac LV tissue sections were settled with acetone for 20 min, taken after endogenous peroxidase blocking with 0.3% H_2_O_2_ solution in methanol. After that, the sections were incubated with NF-ƙB antibodies as primary antibodies [PC137, 1:100 dilution; Calbiochem (EMD Millipore) overnight at 4 °C. At that point, the immunoreactivity was identified using biotinylated secondary antibodies and the avidin-biotin-peroxidase complex was produced. The immunoreactive signal was produced using diaminobenzidine as a substrate for 2 min. The images were taken with the Meiji microscope. The percentage area of NF-ƙB proteins was estimated by using the Image J software package. The percentage area of NF-ƙB was calculated (sum of the total damaged area/total slice area) x100 (39, 40).

***Histopathological assessment of aorta tissue***

The aorta tissues were isolated and preserved in a 10% formalin solution. Sections were stained with hematoxylin (H) and eosin (E) solutions, and observations were analyzed under a Meiji microscope. The Image J software package was used to calculate the plaque percentage area of atheromatous plaques. The atheromatous percentage plaque area was determined as (sum of total accumulated plaque area/total slice area) x 100 (39, 40).

***Statistical analysis***

All data (n=6 per group) were represented as mean ± standard error of the mean (SEM) GraphPad Prism software (version 5.00) was used to compare all of the results statistically. Significant differences of weekly body weight were examined by two-way analysis of variance (ANOVA) followed by Bonferroni *post hoc* tests and other parameters’ significant differences were examined by one-way ANOVA followed Tukey’s multiple comparison test. At *P*<0.05, differences in results were considered statistically significant.

## Results

***Molecular docking analysis***

**Figure 1:**
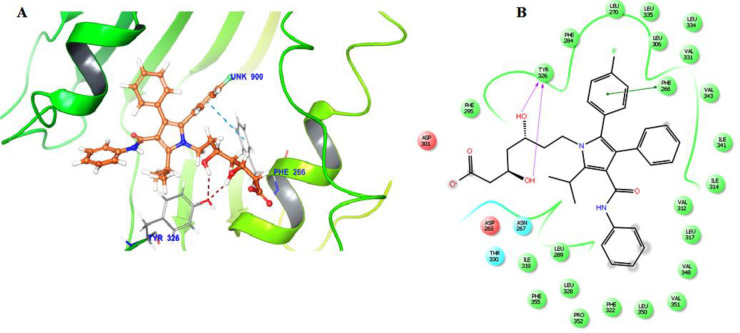
The molecular docking of atorvastatin against TLR2 protein (A) The binding mode of atorvastatin (brown) is depicted, and key residues are marked with grey sticks. (B) A log plot of atorvastatin is displayed in the active site of the TLR2 protein receptor

The molecular docking of atorvastatin was carried out with TLR2 Protein. It has been already reported that TLR2 possesses leucine-rich repeats sequence (LRRs), in its catalytic domain having residues Phe314, Phe312, Gln316, Gly313, Val311 of TLR1, and Leu324, Leu350, Phe325, Phe349, Tyr376, Tyr323 which primarily contributes to the active site of TLR2/TLR1 (41, 42). Tyrosine residues in TLR2 play a crucial role in the interaction of staphylococcal superantigen-like proteins (SSL3) and TLR2. Tyr326 contributes to the inhibitory potency of SSL3 to inhibit TLR2 (43). In the present docking study, results reveal that atorvastatin showed interaction in the catalytic domain, i.e., Phe266 and Tyr326 against TLR2. The atorvastatin and TLR2 complex attained a docking score (-11.545). The benzene ring of atorvastatin formed a pi-pi bond with the phenyl ring of Phe 266, along with a hydrogen bond of two OH groups with Tyr 326 (Figure 1).

***Atorvastatin improved the food and water intake daily in HFD and Zym treated mice***

**Figure 2:**
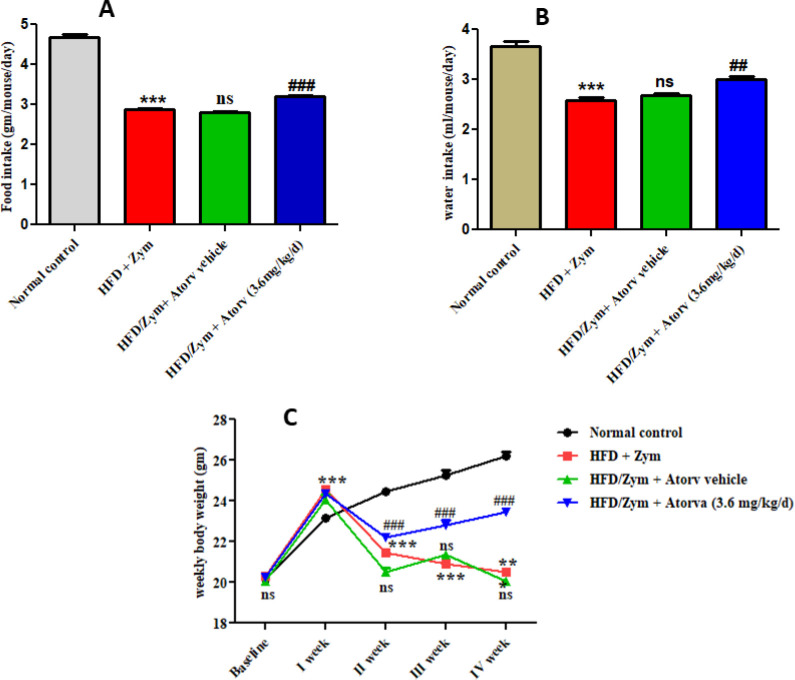
Representative figures showing the effect of atorvastatin on (A) Daily food intake, (B) Daily water intake, and (C) Weekly body weight in HFD and Zym-induced vascular inflammation in C57BL/6 mice.***P<0.001 versus normal control group; ### *P*<0.001, ## *P*<0.01, and ns *P*>0.05 versus HFD+Zym group. Data in the graph are provided as mean ± SEM (n =6)

HFD: High-fat diet; Zym: Zymosan A

It was found that administration of HFD together with Zym, i.e., group II, produced a significant (*P*<0.001) fall in daily food and water intake in C57BL/6 mice when compared with the normal standard chow-diet fed mice, i.e., group I. Treatment with atorvastatin (3.6 mg/kg/day) along with HFD and Zym showed a significant (*P*<0.001), (*P*<0.01) rise in daily food intake and daily water intake, respectively when compared with HFD and Zym treated group. Administration of atorvastatin vehicle, i. e., group III, along with HFD and Zym resulted in non-significant (*P*>0.05) changes in daily food and water intake when compared with the HFD and Zym treated group (Figures 2A, B).

***Effect of atorvastatin on weekly body weight in HFD and Zym treated mice***

In group II, when mice were fed HFD alone in the first week, it was observed that there was a significant (*P*<0.001) elevation in body weight in the first week but, after administration of Zym on the 8^th^ day (80 mg/kg) single IP injection and feeding HFD subsequently for 30 days, a significant (*P*<0.001) loss in body weight was observed when compared with the normal control group, i.e., group I. Administration of atorvastatin (3.6 mg/kg/day), i.e., group IV, along with HFD and Zym showed a significant (*P*<0.001) elevation in body weight as compared with HFD and Zym administered group (Figure 2C). 

***Atorvastatin treatment improved serum lipids levels in HFD and Zym treated mice***

Mice treated with HFD along with Zym, i.e., group II observed a significant (*P*<0.001) increase in serum TC, LDL-C, VLDL, TG levels and a significant (*P*<0.001) decrease in serum HDL-C levels. Administration of atorvastatin (3.6 mg/kg/day) along with HFD and Zym produced a significant (*P*<0.001) reduction in TC, TG, VLDL, and LDL-C levels when compared with the HFD and Zym treated mice. However, administration of atorvastatin vehicle in group III, along with HFD and Zym, resulted in no significant differences (*P*>0.05) in the serum lipid levels when compared with group II (Table 1).

***Atorvastatin reduced atherogenic index (AI) and coronary risk index (CRI)*** ***in HFD and Zym treated mice***

It was observed that there was a significant (*P*<0.001) increase in AI and CRI in HFD and Zym treated mice when compared with the normal control group. When the animals were administered atorvastatin (3.6 mg/kg/day) along with HFD and Zym, it was found that drug treatment significantly (*P*<0.001) reduced HFD and Zym-induced increase in AI and CRI when compared with HFD and Zym administered group (Table 1).

***Atorvastatin reduced hepatic LDLR degradation in HFD and Zym treated mice***

**Figure 3:**
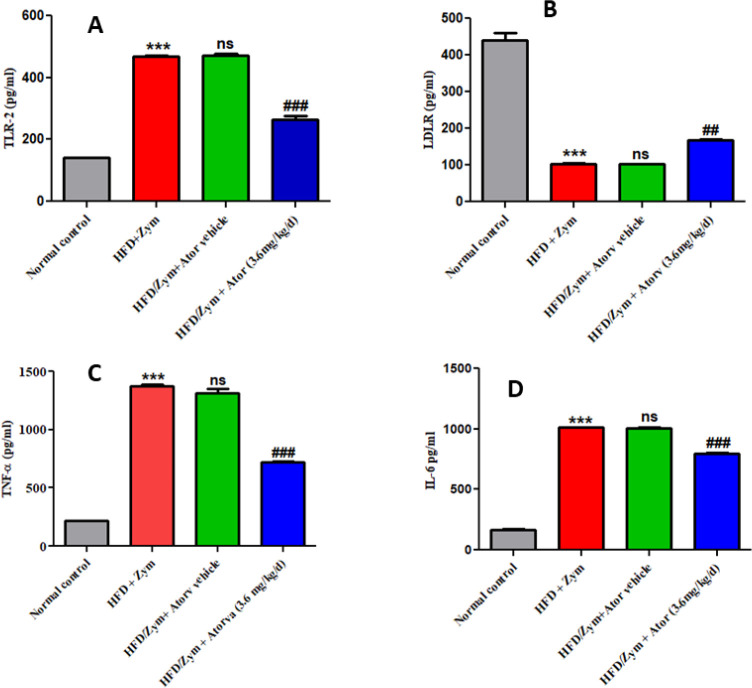
Representative figures showing the effect of atorvastatin on (A) TLR2 levels in aorta tissue, (B) LDLR levels in hepatic tissue, (C) TNF-α levels in serum, and (D) IL-6 levels in serum of HFD and Zym-induced vascular inflammation in C57BL/6 mice. ***P<0.001versus normal control group; ### *P*<0.001, ## *P*<0.01, and ns* P*>0.05 vs HFD+Zym group. The graph's data are provided as mean ± SEM (n =6)

HFD: High-fat diet; Zym: Zymosan A; TLR: Toll-like receptor

In the HFD and Zym treated mice, i.e., group II, it was observed that there was a significant reduction (*P*<0.001) in the LDLR protein levels when compared with the normal control group, i.e., group I. Treatment with atorvastatin (3.6 mg/kg/day), i.e., group IV, together with HFD and Zym observed a significant (*P*<0.01) increase in hepatic LDLR protein when compared with the group II. Administration of atorvastatin vehicle, i.e., group III, along with HFD and Zym produced no significant (*P*>0.05) changes when compared with HFD and Zym administered mice, i.e., group II (Figure 3B).

***Atorvastatin*** ***inhibited HFD and Zym induced aortic TLR2 levels***

In group II stimulation with HFD and Zym led to robust (*P*<0.001) increases in aortic TLR2 levels when compared with the normal control mice, that is, group I. When animals were treated with atorvastatin (3.6 mg/kg/day), i.e., group IV, along with HFD and Zym, it was observed that these treatments significantly (*P*<0.001) down-regulated the aortic TLR2 levels when compared with the HFD and Zym administered mice, i.e., group II (Figure 3A).

***Atorvastatin inhibited HFD and Zym induced NF-*** ƙ***B levels and percentage in the heart LV***

**Figure 4:**
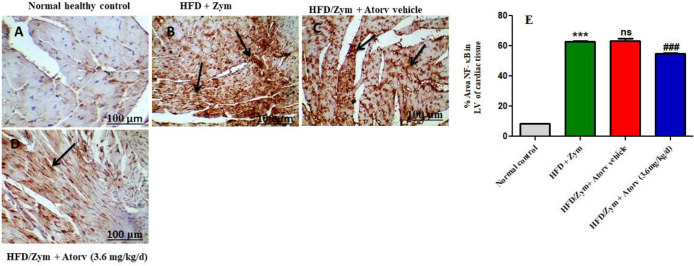
Immunohistochemical evaluation of NF-ƙB in heart LV sections is highlighted by black arrow (brown stain) in cardiomyocytes. [Scale bar - 100 μm]: (IHC, 40X) (n=6) (A) Normal control(sterile PBS ; Zym vehicle), (B) HFD (30 days) and Zym (80 mg/kg IP , single injection on 8th day), (C) HFD/Zym + atorvastatin vehicle (0.5% CMC ), (D) HFD/Zym+ atorvastatin (3.6 mg/kg/day, p.o.), and (E) percentage area of NF-kB was calculated (sum of the total damaged area /total slice area) x100, as assessed from the cardiac sections by Image J software. ***P<0.001 versus normal control group; ###*P*<0.001 and ns *P*>0.05 versus HFD+Zym group. Data in the graph are provided as mean ± SEM (n =6)

HFD: High-fat diet; Zym: Zymosan A

The results demonstrated that HFD and Zym administered mice (group II) had significantly higher levels of NF-ƙB in cardiomyocytes. However, when atorvastatin (3.6 mg/kg/day) was given along with HFD and Zym, the levels and percentage area of NF-ƙB were reduced (Figure 4).

***Atorvastatin lowered*** ***the levels of serum inflammatory mediators, TNF-α and IL-6, in HFD and Zym treated mice***

 In HFD and Zym administered mice (group II), it was observed that there was a substantial increase (*P*<0.001) in the levels of TNF-α and IL-6 when compared with the normal control group. When animals were administered atorvastatin (group IV) along with HFD and Zym, it was observed that there was a significant (*P*<0.001) decreased in TNF-α and IL-6 levels, when compared with HFD and Zym administered mice. Atorvastatin vehicle administration in group III, along with HFD and Zym, resulted in non-significant differences (*P*>0.05) in the levels of serum TNF-α and IL-6 when compared with group II (Figures 3C, D).

***Atorvastatin ameliorated*** ***HFD and Zym induced histopathological changes ***

**Figure 5:**
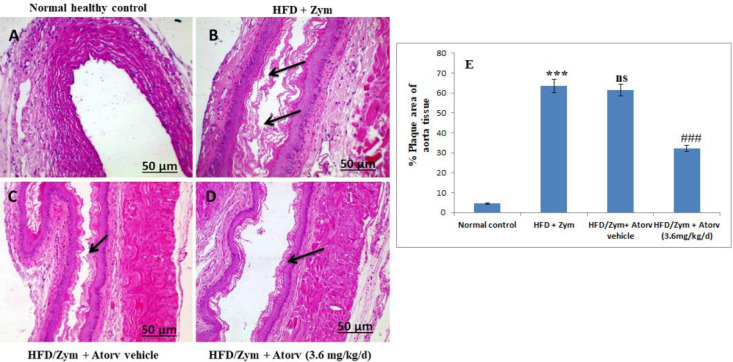
Atorvastatin effect in ameliorating HFD and Zym induced pathological changes in aorta tissue of C57BL/6 mice. Photomicrographs of distinct groups of aorta tissues (H and E staining, 10×) [Scale bar - 50 μm]. The black arrow showed plaque accumulation. (A) Normal control (sterile PBS; Zym vehicle), (B) HFD (30 days) and Zym (80 mg/kg, IP single injection on 8th day), (C) HFD/Zym + atorvastatin vehicle (0.5% CMC), (D) HFD/Zym+ atorvastatin (3.6 mg/kg/day, p.o.), and (E) Atheromatous plaque percentage area was determined as (sum of the total accumulated plaque area / total slice area) x 100, as assessed from the aorta sections by Image J software. ***P<0.001versus normal control group; ###*P*<0.001 and ns *P*>0.05 versus HFD+Zym group. Data in the graph are provided as mean ± SEM (n =6)

HFD: High-fat diet; Zym: Zymosan A

As shown in Figure 4, the aortic tissue in the normal control group represented normal structure without histopathological changes. Mice treated with HFD and Zym for 30 days showed a significant (*P*<0.001) increase in the plaque deposition area percentage in the innermost layer, i.e., intima layer of the aortic tissue, which leads to the narrowing of the aortic wall, when compared with the normal control group. Administration of atorvastatin (3.6 mg/kg/day) in group IV, along with HFD and Zym, resulted in a significant (*P*<0.001) decrease in atheromatous plaque area percentage when compared with the HFD and Zym administered group (Figure 5).

TLR: Toll-like receptor

**Table 1:** Atorvastatin effect on serum lipid levels in HFD and Zym induced vascular inflammation in C57BL/6 mice

**Groups**	**TC ** **(mg/dl)**	**TGs (mg/dl)**	**HDL (mg/dl)**	**LDL (mg/dl)**	**VLDL (mg/dl)**	**AI** **= (LDL-C/HDL-C)**	**CRI** **=(TC/HDL-C)**
Normal control	116 ± 0.33	69.4 ± 0.36	47. 6± 0.13	54.5 ± 0.32	13.88 ± 0.07	1.14 ± 0.007	2.43 ± 0.006
HFD+Zym	254 ± 2.32^a^	263 ± 0.37^a^	15.1 ± 0.90^a^	186 ± 2.34^a^	52.66 ± 0.07^a^	12.29 ± 0.18^a^	16.77 ± 0.20^a^
HFD/Zym+Atorv vehicle	252.8 ± 0.38^ns^	261.5 ± 0.53^ns^	15.7 ±0.30^ ns^	184.7 ± 0.49^ns^	52.8 ± 0.10^ ns^	11.7 ± 0.02^ ns^	16.05 ± 0.25^ ns^
HFD/Zym+Atorvastatin(3.6)	214 ± 0.10^b^	148 ± 0.93^b^	29.6 ± 0.16^b^	154 ± 0.42^b^	29.56 ± 0.35^b^	5.21 ± 0.04^b^	7.208 ± 0.04^b^

HFD: High-fat diet; Zym: Zymosan A; Atorv: Atorvastatin; TC: Total cholesterol; TGs: Triglycerides; HDL-C: High-density lipoprotein-cholesterol; LDL: Low-density lipoprotein-cholesterol; VLDL: Very-low-density lipoprotein-cholesterol; AI: atherogenic index; CRI: coronary risk index. Data are provided as mean ± SEM (n = 6 animals per group)

a*P*<0.001versus normal control group; b*P*<0.001 and ns *P*>0.05 versus HFD+Zym group

## Discussion

Accumulated evidence suggests a direct link between inflammation and arterial disease. The inflammatory response in vascular walls plays a crucial role in the development of arterial disease until clinical signs including dyslipidemia, atherosclerosis, myocardial infarction, and stroke appear (44). Therefore, inhibiting the vascular inflammatory reactions is a potential strategy for preventing cardiovascular events and their associated comorbid conditions (15). The present investigation explored the role of atorvastatin in ameliorating HFD and Zym-induced vascular inflammation and their potential mechanism. 

In the present investigation, we observed that atorvastatin suppressed the vascular inflammatory response by modulating the TLR2/NF-ƙB signaling pathway, reduced release of pro-inflammatory cytokines, decreased plaque accumulation, and improved impaired lipid profile by rescuing the degradation of hepatic LDLR, induced by HFD and Zym administration.

 In the current study, it was observed that oral intake of a high caloric diet in the form of HFD alone in the first week, i.e., initial 7 days without Zym in HFD and Zym group mice significantly increased weekly bodyweight, daily food, and water intake when compared with normal standard chow diet-fed mice. However, administration of Zym (80 mg/kg, single IP injection) on the 8^th^ day together with HFD for another 23 days (total 30 days of HFD feeding) induced acute arterial inflammation in mice, resulting in a substantial decrease in weekly bodyweight and daily food and water intake when compared with the normal standard chow diet-fed group. Our results are in line with the findings of Malik and coworkers (8) who found that administration of Zym to C57BL/6 mice caused inflammation, which led to a catabolic state with an 8% decrease in food consumption, and a 5–7% decrease in body weight in the first 24 hr. Yuan *et al*. (15) also found that rats treated with HFD and Zym were less active and had lower food and water intake for two consecutive weeks.

Further, the present study found that treatment with atorvastatin along with HFD and Zym increased weekly bodyweight and daily food and water intake when compared with the HFD and Zym treated group but not the normal standard chow diet-fed group. Our findings support the findings of Liu *et al*. (7) wherein they reported that curcumin treatment reversed the decrease in body weight due to Zym administration in C57BL/6 mice.

We tested the predicted binding of atorvastatin with TLR2 receptor protein using molecular docking to further investigate whether atorvastatin modulates the TLR2 protein in the current research. The docking study discovered that atorvastatin interacts with TLR2 protein through active binding residues Phe266 and Tyr326. A pi-pi bond was formed between the benzene ring of atorvastatin and the phenyl ring of Phe 266, as well as a hydrogen bond between two OH groups with Tyr 326. The atorvastatin-TLR2 complex received a docking score (-11.545). As a result, the current study reported that atorvastatin has a TLR2 binding affinity. Therefore, the present study confirmed that atorvastatin could modulate the expression of TLR2 protein. Our results corroborate the findings of another study (45) wherein they reported the binding interaction of chitin and TLR2 through docking**. **Authors found that chitin binds with TLR2 through the active sites Val322, Phe296, Phe299, Ser320, Thr309, and Leu302 having two hydrogen bonds.

TLR2 are pattern recognition receptors of innate immunity that are highly expressed in endothelial cells placed at regions of susceptibility to atherosclerosis, such as the aortic arch (46). There is growing evidence that TLR2 is the major initiator of inflammation and promotes atherosclerosis (47). Engagement of TLR2 on immune and resident vascular cells can affect arterial inflammatory response that leads to inflammatory pathway activation, release of inflammatory mediators, and altered endothelial function and has been implicated in the development of almost all metabolic and atherosclerotic diseases (48). Atorvastatin’s mechanism against the TLR2 pathway in HFD and Zym-induced vascular inflammation is as yet not explored. Therefore, to find out whether the anti-inflammatory activity of atorvastatin is mediated by the TLR2 signaling pathway, we evaluated the TLR2 expression in the aorta of mice. In the present investigation, it was found that administration of HFD along with Zym enhanced arterial inflammation by increasing the aortic TLR2 levels. As a result, the area of atheromatous plaque in the aorta increases, as confirmed by the histological investigation. Treatment with atorvastatin along with HFD and Zym decreased TLR2 levels and plaque accumulation in mice aorta. Our findings are in line with another study (49) wherein they demonstrated that exogenous administration of synthetic TLR2 agonist activates TLR2 in the aorta and enhances atherosclerotic plaque accumulation and plaque media ratio in ApoE-/- atherosclerotic mice. Furthermore, research carried out by other researchers (50) reported that deficiency of TLR2 reduces foam cell production in lesion-prone regions of the aorta in ApoE/ mice.

Researchers reported that TLR2 triggered the inflammatory response by activation of NF-ƙB (2). Transcription factor NF-kB is considered a major intracellular inflammatory pathway that mediates most of the vascular inflammatory response (51, 52). It is normally found in the cytoplasm, but when activated, it translocates into the nucleus. Then nucleus-located NF-kB will trigger and release various cytokines genes, such as TNF-α and IL-6 which lead to atherosclerotic plaque instability and rupture (15, 53). As a result, in the current study we evaluated the levels of cardiac NF-ƙB in HFD and Zym induced vascular inflammation in C57BL/6 mice. The present research work indicates that treatment with HFD together with Zym increased the cardiac NF-kB levels. However, treatment with atorvastatin decreased NF-kB levels in cardiomyocytes, which may be mediated by down-regulation of TLR2. The result confirmed that atorvastatin could attenuate the HFD and Zym-induced vascular inflammation via inhibiting the NF-ƙB levels. These findings are in line with the findings of Yuan and colleagues (15) who found that administering Wistar rats HFD and Zym (20 mg/kg, IP, single injection every 3 days for 2 weeks) for 9 weeks resulted in elevated NF-ƙB expression, which was reversed by Panax notoginseng saponin treatment. Our results are also corroborated with the findings of another study (40) wherein they found that administration of interleukin-18 in mice induced vascular inflammatory response which increased the NF-ƙB expression in cardiomyocytes. Also, researchers (54) reported that suppression of TLR2 and NF-kB levels alleviates atherosclerotic inflammation. Thus, the findings of our study revealed that the anti-inflammatory role of atorvastatin may be attributed to modulation of the TLR2/NF-ƙB signaling pathway against HFD and Zym-induced vascular inflammation in C57BL/6 mice.

According to previous research, activation of TLR2 promotes the release of inflammatory mediators such as TNF-α and IL-6 via NF-kB signaling, resulting in vascular wall dysregulation (2). Researchers reported that atorvastatin inhibits secretion of IL-1β and TNF-α induced by lipopolysaccharide (55, 56). Consistent with these previous studies, the results of the current study, showed that the HFD and Zym administered group showed an increase in the levels of TNF-α and IL-6 which was significantly reduced by atorvastatin treatment.

Vascular inflammation mediates distinct changes in lipid and lipoprotein metabolism (14). Inflammation and hypercholesterolemia are related to a critical condition in which an accumulation of cholesterol in the artery wall triggers an inflammatory response, which increases cholesterol deposition and exacerbates vascular inflammation (57). Collected evidence suggests that inflammation affects cholesterol homeostasis by causing hepatic LDLR degradation intracellularly. Degradation of the LDLR is closely related to cholesterol aggregation and foam cell development (58, 59). A study (14) reported that administration of HFD for 7 days and Zym (80 mg/kg, *IP,* single injection) in C57BL/6 mice promotes inflammation, which results in a decrease in LDLR and increases LDL-C levels. Interestingly, our results showed that treatment of HFD and Zym reduced hepatic LDL receptor levels, resulting in a decrease in circulating LDL-C clearance and accounting for the increase in serum LDL-C levels. Besides this, we also found an alteration in the lipid metabolic profile (significant increase in serum TC, LDL-C, VLDL, and TG levels and decrease in serum HDL-C levels) in C57BL/6 mice. Further, treatment with atorvastatin along with HFD and Zym significantly increased the hepatic LDLR levels which resulted in decreased LDL-C levels. In addition, atorvastatin treatment decreased TC, VLDL, and TG levels and also decreased AI and CRI, and significantly increased HDL-C levels as compared with HFD and Zym only administered mice.

The above findings were confirmed by the results of the histological analysis showing that the HFD and Zym administered group had increased atheromatous plaque area in aorta tissue. Atorvastatin treatment showed a decrease in the atheromatous plaque area in aorta tissue.

## Conclusion

Our study provides evidence that HFD together with Zym induces vascular inflammation in C57BL/6 mice. Atorvastatin (3.6 mg/kg/d) treatment can effectively attenuate the vascular inflammation induced by HFD and Zym in C57BL/6 mice by significantly reducing the lipid levels including TC, LDL, VLDL, and TG levels and also decreased AI and CRI and increased HDL-C levels. Furthermore, atorvastatin treatment suppresses the vascular inflammatory response via inhibiting the expression of TLR2, NF-ƙB, LDLR, TNF-α, and IL-6 and reduces plaque accumulation in the aorta. The investigation thus clearly exhibits anti-inflammatory and anti-atherosclerotic effects of atorvastatin against HFD and Zym induced vascular inflammation.
